# Development of a Combined Risk Factors Prediction Model for Esophageal Squamous Cell Carcinoma: A Secondary Analysis of the Linxian Nutrition Intervention Trial Cohort

**DOI:** 10.21203/rs.3.rs-10027456/v1

**Published:** 2026-07-09

**Authors:** Huan Yang, Jingyi Shi, Jianbing Wang, Jinhu Fan, Shaokai Zhang, Yin Liu, Huifang Xu, Vikash Sewram, Jiangong Zhang, Fanghui Zhao, Youlin Qiao, Christian C. Abnet

**Affiliations:** Cancer Hospital of Chinese Academy of Medical Sciences; Chinese Academy of Medical Sciences & Peking Union Medical College; Children’s Hospital of Zhejiang University; Cancer Hospital of Chinese Academy of Medical Sciences; Henan Cancer Hospital; Henan Cancer Hospital; Henan Cancer Hospital; Stellenbosch University; Henan Cancer Hospital; Cancer Hospital of Chinese Academy of Medical Sciences; Chinese Academy of Medical Sciences & Peking Union Medical College; National Cancer Institute

**Keywords:** Esophageal squamous cell carcinoma, risk factors, risk prediction, risk score

## Abstract

**Background:**

Early screening and detection is essential to reduce morbidity and mortality in esophageal cancer (EC), particularly for individuals at high risk. To reduce the burden and high costs of whole-population screening, we developed a risk score model for individualized risk assessment of esophageal squamous cell carcinoma (ESCC) incidence.

**Methods:**

The study was conducted using the Linxian Nutrition Intervention Trial cohort. Cox regression and the points system method were used to build a score-based model for ESCC risk prediction. The receiver operating characteristic (ROC) curve and calibration curve were used to examine the distinction and calibration of the models.

**Results:**

A total of 29,408 participants were included in final analysis. During the 10-year follow-up period, 1386 ESCC new cases were identified. Cox regression showed that increasing age, smoking, family history of esophageal cancer, dysphagia, fresh vegetable consumption (≤ 1 time/day), low body mass index (BMI < 18.5kg/m^2^), not drinking tap water (versus untreated natural water), and tooth loss were independent risk factors of ESCC incidence. The risk score based on 8 risk factors ranged from 0 to 59 points. Compared to subjects with a risk score < 20 points, the ESCC incidence risk increased by 201% for 20 to 39 points and 615% for score of over 39 points. The area under the curve (AUC) value of the risk score estimating ESCC incidence within 3 years was 0.70 (95% CI: 0.67–0.72).

**Conclusions:**

Our model effectively stratified the risk of ESCC, demonstrating a potential application in high-risk population identification and ESCC prevention.

## Background

Esophageal cancer (EC) is a common gastrointestinal cancer,^1, 2^ ranking 11th in cancer incidence and 7th in cancer mortality.^3^ The 5-year survival rate of patients with the middle-advanced stage EC is only 15 to 25%,^1^ whereas it may reach 80% or higher in early-stage patients.^4^ This suggested that early detection of EC cancer reduce the morbidity and mortality due to EC. Chromoendoscopy, biopsy of unstained lesions, and histopathological examination is considered the gold standard for esophageal squamous cell carcrioma (ESCC) early detection.^5^ However, due to the invasiveness and high cost of endoscopy, it may be more cost-effective to screen only individuals estimated to be at high-risk rather than the whole population.^6^ Therefore, seeking effective and convenient risk identification tools is urgently needed in ESCC prevention.

Previous studies have provided a comprehensive overview of the risk factors associated with EC and may be useful in identifying high-risk individuals.^5, 7–9^ To analyze the effect of combined risk factors, some researchers have explored risk prediction models. However, most of the models were developed in western countries where esophageal adenocarcinoma (EAC) was the main subtype of EC.^5^ Considering there are notable differences in the natural progression and risk factors among different subtypes, risk prediction models for esophageal squamous cell carcinoma (ESCC) are still needed from large-scale population studies. China has a large EC burden,^3^ where 86% of EC cases were ESCC.^10^ Therefore, some studies have established risk prediction models of ESCC incidence or mortality based on Chinese populations.^4, 11–13^ Yet, most of these studies were based on screening population rather than the general population. In addition, few of these studies have considered the timeliness of single-time collection on risk factors in the ESCC incidence prediction.

In this study, we focused on a general population cohort with high ESCC incidence in Linxian, Henan Province, China.^14^ Based on the cohort, this study aimed to establish a risk score for ESCC, which may assess the risk of ESCC incidence for populations in high-incidence areas and guide primary and secondary prevention of ESCC.

## Methods

### Study Population

**Figure 1:**
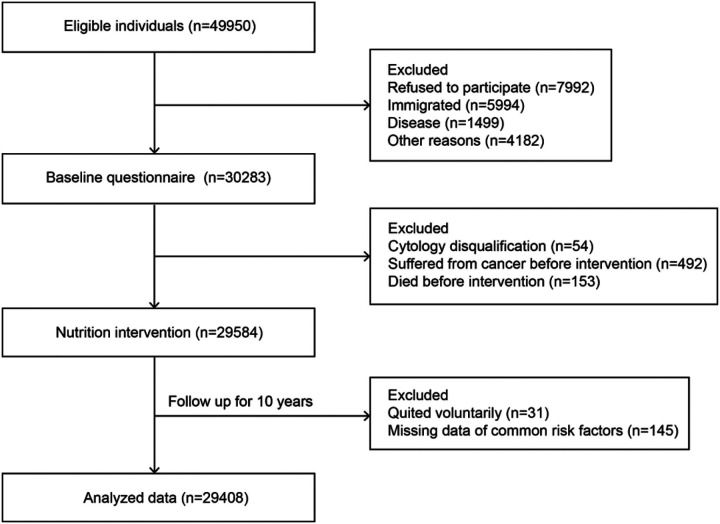
Flow diagram of the study population Fig 1 presents the inclusion and exclusion process for participants in the final analyses.

This study was a secondary analysis based on the Linxian General Population Trial cohort initiated in 1985.^15^ Detailed information have been described in previous studies.^16, 17^ The inclusion criteria were as follows: (a) Healthy individuals aged 40 to 69 years; (b) Lived in one of the four communes in Northern Linxian (Yaocun, Rencun, Donggang, and Hengshui); (c) Participated in the study voluntarily and signed the informed consent form. The exclusion criteria were as follows: (a) Had cancer or other malignancies previously; (b) Regularly took vitamins and minerals supplements. Finally, a total of 29,584 eligible subjects were recruited in this cohort and received daily multivitamins and minerals supplements or placebo according to a 2^4^ fractional factorial design.^18^ The intervention began in March 1986 and ended in May 1991, after which all participants were prospectively followed up until now. Finally, 29,408 patients were included in the study (Fig. 1). This study was approved by the Institutional Review Boards of the Cancer Hospital Chinese Academy of Medical Sciences (CHCAMS) and the US National Cancer Institute (NCI) (No. 22/445 – 367).

### Collection of the Baseline Risk Factors

Baseline characteristics were collected by trained investigators in 1985 using a structered questionnaire, including demographic information, lifestyle, family history of cancer, disease history, and dietary habits, etc. Based on previous evidence of ESCC risk factors,^5, 7 8^ we selected 14 possible risk factors of ESCC, including age at baseline, sex, body mass index (BMI, kg/m^2^), smoking status, alcohol drinking, family history of EC, fresh vegetable consumption, fresh fruit consumption, hot drink consumption, pickled vegetable consumption, drinking tap water, tooth loss, dysphagia, and history of digestive diseases. Smoking was defined as smoking cigarettes, or using hookah or pipes at least weekly for more than 6 months. Alcohol drinking was divided into consuming alcoholic beverages (such as wine, beer, or liquor) daily or not in the past 12 months.^5, 7 8 10^ Family history of EC was defined positive if subject had one or more first-degree relatives diagnosed with EC. The intake frequency of fresh vegetables, fresh fruits, hot drinks, and pickled vegetables were recorded and transformed to categories, according to whether participants were exposed to these factors weekly or daily. Drinking tap water was defined as having tap water installed as drinking water source rather than drinking well water or mountain spring water.^19^ Tooth loss was defined positive if the participant exhibited missing teeth or indicated for extraction, such as mobile teeth, severely damaged teeth, root stumps, or the presence of a fixed or removable prosthesis.^20, 21^ Dysphagia was defined as difficulty in swallowing. A history of digestive diseases was defined as having a history of diseases, including but not limited to, chronic gastritis, gastric ulcers, and duodenal ulcers, etc. In addition, a brief physical examination was conducted by well-trained medical workers. Body height and weight were measured without shoes according to a standard protocol. BMI was calculated by dividing body weight (kg) by the square of body height (m^2^). Considering underweight (BMI < 18.5 kg/m^2^) had significant association with the risk of ESCC,^22^ we divided BMI into two groups using 18.5 as the cut-off value (BMI < 18.5kg/m^2^; BMI > 18.5kg/m^2^).

### Follow-up and Outcome Assessment

Participants were prospectively followed up monthly and censored at the date of death, date lost to follow-up, or the last follow-up date for analysis (Nov 2023). The main outcome was ESCC incident cases (ICD10:C15). To ensure the accuracy of outcome, relevant diagnostic materials such as case records, biochemical results, ultrasound, X-ray, endoscopy, surgery reports and pathology or cytology slides were verified by a panel of American and (or) senior Chinese experts. Considering that the efficiency of one-time collection of epidemiological risk factors to predict the onset of ESCC may decline over time, we constructed the prediction model using 10-year follow-up data after baseline (from March 1986 to February 1996).

## Statistical Analysis

Participants from all the intervention groups of the trial cohort were included in this study, assuming no interaction between randomization group and the influence of other predictors on ESCC risk. Differences of demographic characteristics at baseline between new ESCC cases and normal participants were compared using t-test for continuous variables and Pearson’s Chi-square test for categorical variables.

We included 14 risk factors mentioned above as well as the nutrition intervention group in the univariate Cox regression model, and variables with *P values* less than 0.1 were included in the multivariate Cox regression model. We used backward stepwise regression and the Akaike Information criterion (AIC) was used in model selection. Variables with a *P value* less than 0.05 in the multivariate regression were considered independent risk factors of ESCC, and multivariate regression model that included independent risk factors were considered as benchmark model. A curve was plotted with follow-up time as the abscissa and the benchmark model’s C-indexes as the ordinate, to evaluate the model’s performance over time.

We developed a risk score using a “points system” method,^23^ which was applied to simplify the benchmark model as a risk score system. The detailed procedures were described in Supplementary Method[see Additional file 1]. The final risk score was included in the Cox regression models again to calculate the hazard ratios (HRs) and 95% confidence intervals (95%CIs), treated as both continuous and categorical variables (based on the tertiles). Subgroup analysis was performed to assess whether the association varied by sex. The cumulative incidence rates were estimated using the Kaplan-Meier method, and Log-rank tests were used to examine the difference among score categories. Restricted cubic splines (5 knots) of risk score were plotted for graphical assessment.

The discrimination ability of both the benchmark model and the score-based model were evaluated by the area under the receiver operating curve (AUC) with 95%CI, using 5-fold cross-validation as internal validation. Calibration curves were plotted to evaluate the goodness of fit. The structure of the article followed the STROBE guideline for cohort studies. The threshold for significance was *P* < 0.05 in 2-sided tests. All statistical analyses were performed in R version 4.3.1.

## Results

### Baseline Characteristics

After excluding individuals lost to follow-up and with missing risk factors (176 excluded), a total of 29,408 participants were included in our study. The mean baseline age was 51.9 ± 8.9, and women accounted for 55.4% of volunteers. A total of 378, 697, and 1386 ESCC new cases were identified during the 3, 5, and 10-year follow-up period, respectively. According to Table 1, ESCC cases were more likely to be older, males, smokers, had lower BMI, higher hot drink consumption, higher vegetable consumption, not drinking tap water, and had higher percentage of family history of EC in first-degree relatives, missing teeth and symptoms of dysphagia.

### Benchmark Model Based on Cox Regression

Table 2 summarizes the HRs and 95% CIs of the associations between common risk factors and ESCC incidence. The multivariate Cox regression model (benchmark model) showed that age (for an increase of 1 year), smoking, family history of EC, BMI<18.5 kg/m^2^, not drinking tap water, tooth loss, dysphagia, and fresh vegetable consumption ≤ 1 time/day were the independent risk factors for ESCC. The ability of the benchmark model assessing the risk of ESCC incidence decreased slightly over time, with the best estimation within 2.8 years (Figure S1).

Table 3 lists the AUCs of the benchmark model in estimating the ESCC incidence during 3-, 5-, and 10-year follow-up period. The model had the best ability to assess the ESCC incidence within 3-year follow-up with an AUC of 0.71 (95%CI: 0.68–0.73). Similar discrimination ability was observed in the the 5-fold cross-validation (AUC = 0.70, 95%CI: 0.68–0.73). The calibration curves were all approximately diagonal and demonstrated good calibration (Figure S2).

### Risk Score for Predicting ESCC Incidence

Based on the coefficients of the benchmark model, we established a risk score using “points system” method (Table S1). The final score ranged from 0 to 59. Higher scores indicated a higher risk of developing ESCC. Based on the tertiles of the score, patients were categorized into three groups: 18,652 (63.4%) patients with scores < 20 points, 10,353 (35.2%) patients with scores between 20 and 39 points, and 403 (1.4%) patients with scores > 39 points. The cumulative ESCC incidence rates of the three groups within 3 years were 0.7%, 2.2%, and 4.7%. For 3-year ESCC incidence prediction, compared to subjects with a risk score < 20 points, the risk increased by 201% (HR = 3.01, 95% CI: 2.37–3.82) for 20 to 39 points and by 615% (HR = 7.15, 95% CI: 4.29–11.91) for > 39 points. For every one-point increase in score, the risk increased by 7% (HR = 1.07, 95% CI: 1.06–1.09, *P*_*frend*_<0.001) (Table 4). Restricted cubic splines suggested a positive dose-response relationship between the risk score and the risk of ESCC onset (Figure S3). Cumulative incidence curves of ESCC by risk score categories are showed in Figure S4.

Results of subgroup analysis by sex is shown in Table S2. In predicting 3-year ESCC incidence, no interaction was observed between sex and the risk score (*P*_*jnteractjon*_=0.86), and the associations were statistically significant in both men and women (*P*_*frend*_<0.001).

**Figure 2:**
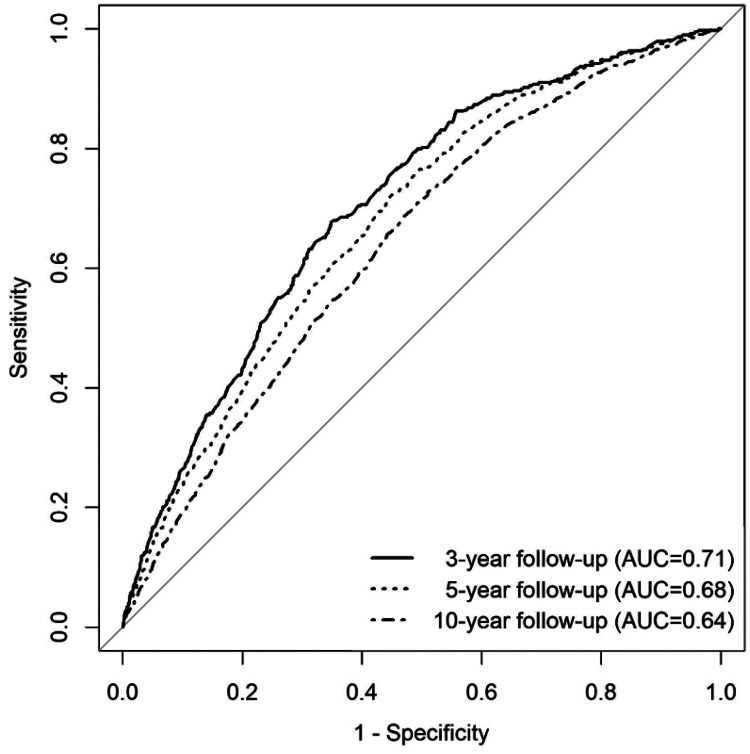
ROC curves for predicting the risk of ESCC within 3, 5 and 10 years Fig 2 presents the receiver operating characteristic (ROC) curves of the model across three follow-up periods: 3 years (solid line, AUC = 0.71), 5 years (dotted line, AUC = 0.68), and 10 years (dashed line, AUC = 0.64).

ROC curves of the risk score for predicting ESCC incidence are shown in Fig. 2. The AUC of the risk score for predicting 3-, 5- and 10-year ESCC incidence were 0.70 (95%CI: 0.68–0.73), 0.68 (95%CI: 0.66–0.70) and 0.64 (95%CI: 0.63–0.66), respectively, which were similar to those of the benchmark model. Table S3 shows the sensitivity and specificity for predicting the 3-year risk of ESCC with different cut-off values, and 20.5 was considered as the best cut-off value, with the sensitivity and specificity of 78.04% and 53.66%, respectively (Table S4).

## Discussion

In this study, we established a predictive risk-scoring model for ESCC incidence. A total of 8 risk factors of ESCC were considered in the model, including age, smoking, family history of esophageal cancer in first-degree relatives, dysphagia, fresh vegetable consumption, BMI, drinking tap water, and tooth loss.

Previous studies have identified risk factors of ESCC^24^. Although the discriminative performance reported by published models was mostly acceptable, the calibration metrics were not adequately reported.^25^ In our study, we established a combined risk score, which can be easy applied without calculator or computer. The comparison of AUC between the benchmark model and the risk scoring model showed similar discrimination ability, indicating that the conversion from the complex model to the score does not result in a loss of predictive power.

Age is a risk factor with the largest effect in our model, possibly due to age-related immune decline and the accumulation of cellular damage.^26, 27^ Tobacco smoking, a Type I carcinogen,^28^ also increases ESCC incidence risk.^29^ Polycyclic aromatic hydrocarbons (PAHs) and tobacco-specific nitrosamines (TSNAs), which have been identified as the likely primary carcinogens in tobacco,^30^ may potentially act upon the esophagus by ingestion, leading to carcinogenesis.^31^

One unexpected finding in our study was that alcohol consumption was associated with lower ESCC incidence risk, although the difference was not statistically significant. Studies based on Iranian population^32^ and China population^20^ found the similar results. One possible reason is that in 1985, alcohol consumption in Linxian was rare, the total amount consumed was low, and might be a signal for higher socioeconomic status.^33^

Fresh fruits and vegetables are the main sources of minerals, vitamins, antioxidants, and dietary fiber.^34^ High intake of vegetables and fruits may reduce the risk of ESCC.^35^ However, only 7% of the participants in our study consumed fresh fruits more than once a week, which may explain the marginal association between fruit consumption and ESCC incidence. In addition, we found that individuals who drank tap water had a lower risk of ESCC. The association can be attributed to the chemical pollutants such as nitrites and nitrosamines in water,^36^ which have mutagenic, teratogenic, and carcinogenic properties.^37^

Unlike EAC,^38^ overweight or obesity was found to be a protective factor for ESCC.^22^ Similar associations were observed in cohort studies conducted in China and in the Korean population.^39, 40^ One explanation seems to be that underweight individuals with poor nutritional status over the long term may contribute to the development of ESCC. In addition, the confounding of smoking and heavy alcohol assumption, which were associated with BMI,^41, 42^ may have the combined effect on the development of ESCC.

Early-stage EC often presents with subtle symptoms such as dysphagia.^43^ This underlying association makes it a potential risk factor for predicting the incidence of EC. Some studies suggested an association between tooth loss and EC.^44, 45^ One possible reason was that tooth loss may lead to the ingestion of poorly chewed food, which could potentially irritate the esophageal mucosa.^15^ Another hypothesis suggested that tooth loss may disrupt periodontal tissue, leading to the accumulation of oral microorganisms deep within tissues, thereby promoting their growth.^46^

Family history of EC can represent the influence of both environmental and genetic factors.^47^ Results from a case-control study suggested a potential stronger association between genetic factors and ESCC incidence in high-risk areas.^48, 49^ In addition, family members in Linxian share the same risk factors due to long-term residence, which leads to an increasing risk of developing ESCC.^50^

Some ESCC risk factors, such as smoking, may change over time, even if they remain stable in the short term. Thus, attention must be paid to the timeliness of the prediction. Our model has demonstrated the greatest performance for the 3-year prediction. However, the HRs in different score categories all slightly decreased by time, either in all subjects or in subgroups.

Our study has several advantages. Firstly, we used a large-scale dataset with a low attrition rate. Secondly, the “points system” method is easy to understand and apply in public health practices. Thirdly, we evaluated different effects in different time period, and examined the model’s stability by time. What’s more, the baseline variables in our dataset were diverse and well-designed, empowering us to verify multiple risk factors. Finally, the model was set up in a high-incidence area of ESCC, enriching the population data in the field and providing targeted suggestions for the prevention and screening of ESCC in China.

Yet, the AUC of our model, which was constructed using epidemiological variables, was moderate due to the absence of markedly dominant predictors. The similar lifestyle in Linxian population may also present a challenge in distinguishing high-risk populations, consequently impacting the model’s predictive efficacy. Besides, some risk factors such as lifestyles may change overtime and become different with baseline information. Unmeasured factors such as socioeconomic status and physical activity may lead to residual confounding. Finally, although we used cross validation to evaluate the accuracy, our model has not been tested in other populations.

## Conclusions

In summary, we developed an ESCC prediction scoring model based on 8 common and easy-to-get risk factors, which can be applied to predict the incidence of ESCC and contribute to high-risk population identification and precision prevention.

## Supplementary Files

This is a list of supplementary files associated with this preprint. Click to download.

- Additionalfile1.docx

## Figures and Tables

**Table 1:** Baseline characteristics of 29,408 individuals included in the study

Risk Factors	Individuals with ESCC (n = 1386)	Individuals without ESCC (n = 28022)	Total (n = 29408)	*P values* ^a^
Age (years, mean ± SD)	55.5 ± 8.1	51.7 ± 8.9	51.9 ± 8.9	< 0.001
Sex (n, %)				0.003
Female	714 (51.5)	15590 (55.6)	16304 (55.4)	
Male	672 (48.5)	12432 (44.4)	13104 (44.6)	
Smoking status (n, %)				< 0.001
No	877 (63.3)	19706 (70.3)	20583 (70.0)	
Yes	509 (36.8)	8316 (29.7)	8825 (30.0)	
Daily alcohol drinking (n, %)				0.318
No	1076 (77.6)	21428 (76.5)	22504 (76.5)	
Yes	310 (22.4)	6594 (23.5)	6904 (23.5)	
Family history of esophageal cancer in first-degree relatives (n, %)				< 0.001
No	912 (65.8)	20430 (72.9)	21342 (72.6)	
Yes	474 (34.2)	7592 (27.1)	8066 (27.4)	
Drinking tap water (n, %)				0.024
Yes	307 (22.2)	6955 (24.8)	7262 (24.7)	
No	1079 (77.8)	21067 (75.2)	22146 (75.3)	
Tooth loss (n, %)				< 0.001
No	233 (16.8)	7203 (25.7)	7436 (25.3)	
Yes	1153 (83.2)	20819 (74.3)	21972 (74.7)	
Dysphagia (n, %)				0.004
No	1318 (95.1)	27059 (96.6)	28377 (96.5)	
Yes	68 (5.9)	963 (3.4)	1031 (3.5)	
BMI (n, %)				< 0.001
≥ 18.5 kg/m^2^	1284 (92.6)	26572 (94.8)	27856 (84.7)	
< 18.5 kg/m^2^	102 (7.4)	1450 (5.2)	1552 (5.3)	
Fresh vegetable consumption				< 0.001
> 1 time/day	1160 (83.7)	24322 (86.8)	25482 (86.7)	
≤ 1 time/day	226 (16.3)	3700 (13.2)	3926 (13.3)	
Fresh fruit consumption (n, %)				0.121
> 1 time/week	85 (6.1)	2027 (7.2)	2112 (7.2)	
≤ 1 time/week	1301 (93.9)	25995 (92.8)	27296 (92.8)	
Hot drink consumption (n, %)				0.028
≤ 1 time/day	1003 (72.4)	19501 (69.6)	20504 (69.7)	
> 1 time/day	383 (27.6)	8521 (30.4)	8904 (30.3)	
Pickled vegetable consumption (n, %)				0.307
≤ 1 time/week	1333 (96.2)	27092 (96.7)	28425 (96.7)	
> 1 time/week	53 (3.8)	930 (3.3)	983 (3.3)	
History of digestive diseases (n, %)				0.063
No	1237 (89.2)	25425 (90.7)	26662 (90.7)	
Yes	149 (10.8)	2596 (9.3)	2745 (9.3)	
Nutrition intervention group^b^				0.051
Placebo Group	175 (12.6)	3503 (12.5)	3678 (12.5)	
AB Group	190 (13.7)	3501 (12.5)	3691 (12.6)	
AC Group	148 (10.7)	3520 (12.6)	3668 (12.5)	
AD Group	149 (10.8)	3517 (12.6)	3666 (12.5)	
BC Group	179 (12.9)	3496 (12.5)	3675 (12.5)	
BC Group	193 (13.9)	3485 (12.4)	3678 (12.5)	
CD Group	161 (11.6)	3508 (12.5)	3669 (12.5)	
ABCD Group	191 (13.8)	3492 (12.5)	3683 (12.5)	

Abbreviations: ESCC, esophageal squamous cell carcinoma; BMI, body mass index.

a. Analyses were conducted by t-test for continuous variables and Pearson’s Chi-squared test for categorical variables.

b. Subjects received daily multivitamins and minerals supplements or matching placebo according to a 2^4^ fractional factorial design.

**Table 2:** HRs and 95% CIs in univariate and multivariate analyses

Variables	Univariate analysis		Multivariate analysis	
	HR (95%CI)^a^	*P value*	HR (95%CI)^a^	*P value*
Age (years)	1.06 (1.05 to 1.06)^b^	< 0.001	1.05 (1.05 to 1.06)^b^	< 0.001
Sex		< 0.001		-^d^
Female	Ref		-^d^	
Male	1.23 (1.11 to 1.37)		-^d^	
Smoking status		< 0.001		< 0.001
No	Ref		Ref	
Yes	1.42 (1.27 to 1.59)		1.36 (1.22 to 1.51)	
Daily alcohol drinking		0.222		-^d^
No	Ref		-^d^	
Yes	0.92 (0.81 to 1.05)		-^d^	
Family history of esophageal cancer in first-degree relatives		< 0.001		< 0.001
No	Ref		Ref	
Yes	1.38 (1.24 to 1.54)		1.40 (1.26 to 1.57)	
Drinking tap water		0.022		0.036
Yes	Ref		Ref	
No	1.16 (1.02 to 1.32)		1.15 (1.01 to 1.30)	
Tooth loss		< 0.001		0.027
No	Ref		Ref	
Yes	1.79 (1.56 to 2.06)		1.18 (1.02 to 1.38)	
Dysphagia		0.002		0.004
No	Ref		Ref	
Yes	1.46 (1.15 to 1.87)		1.43 (1.12 to 1.82)	
BMI		< 0.001		0.011
≥ 18.5 kg/m^2^	Ref		Ref	
< 18.5 kg/m^2^	1.53 (1.25 to 1.87)		1.30 (1.06 to 1.59)	
Vegetable consumption		< 0.001		0.010
> 1 time/day	Ref		Ref	
≤ 1 time/day	1.27 (1.10 to 1.47)		1.21 (1.05 to 1.39)	
Fruit consumption		0.104		-^d^
> 1 time/week	Ref		-^d^	
≤ 1 time/week	1.20 (0.96 to 1.49)		-^d^	
Hot drink consumption		0.034		-^d^
≤ 1 time/day	Ref		-^d^	
> 1 time/day	0.88 (0.78 to 0.99)		-^d^	
Pickled vegetable consumption		0.363		-^d^
≤ 1 time/week	Ref		-^d^	
> 1 time/week	1.14 (0.86 to 1.49)		-^d^	
History of digestive diseases		0.071		-^d^
No	Ref		-^d^	
Yes	1.17 (0.99 to 1.39)		-^d^	
Nutrition intervention group^c^				
Placebo Group	Ref			
AB Group	1.10(0.89 to 1.35)	0.375	-^d^	-^d^
AC Group	0.85(0.68 to 1.06)	0.148	-^d^	-^d^
AD Group	0.86(0.69 to 1.07)	0.165	-^d^	-^d^
BC Group	1.03(0.84 to 1.27)	0.792	-^d^	-^d^
BC Group	1.11(0.90 to 1.36)	0.335	-^d^	-^d^
CD Group	0.93(0.75 to 1.15)	0.505	-^d^	-^d^
ABCD Group	1.10(0.89 to 1.35)	0.375	-^d^	-^d^

Abbreviations: HR, hazard ratio; CI, confidence interval; BMI, body mass index.

a. HRs of variables with *P values* ≥ 0.1 in univariate analyses and *P values* ≥ 0.05 in multivariate analyses were not shown.

b. HR for age is for an increase of 1 year.

c. Subjects received daily multivitamins and minerals supplements or matching placebo according to a 2^4^ fractional factorial design.

d. variables excluded in multivariable cox regression.

**Table 3:** AUCs and 95%CIs of the benchmark model predicting the risk of ESCC incidence

	AUC (95%CI)		
	3 years	5 years	10 years
Without cross-validation	0.71 (0.68 to 0.73)	0.68 (0.66 to 0.70)	0.64 (0.63 to 0.66)
5-fold cross-validation^a^	0.70 (0.68 to 0.73)	0.68 (0.66 to 0.70)	0.64 (0.63 to 0.66)

Abbreviations: AUC, area under curve; CI, confidence interval; ESCC, esophageal squamous cell carcinoma.

a. Average AUCs of 5-fold cross-validation

**Table 4:** HRs and 95% CIs for the associations between risk score and risk of ESCC incidence

	Risk score categories^a^			Continuous	*P*_*trend*_
	< 20	20–39	> 39		
3-year follow-up					
HR (95%CI)	Ref	3.32 (2.67 to 4.13)	7.50 (4.63 to 12.16)	1.07 (1.06 to 1.08)	< 0.001
Adjusted HR (95%CI)^b^	Ref	3.01 (2.37 to 3.82)	7.15 (4.29 to 11.91)	1.07 (1.06 to 1.09)	< 0.001
5-year follow-up					
HR (95%CI)	Ref	2.76 (2.36 to 3.22)	6.60 (4.59 to 9.48)	1.07 (1.06 to 1.07)	< 0.001
Adjusted HR (95%CI)^b^	Ref	2.42 (2.04 to 2.87)	5.89 (4.03 to 8.63)	1.06 (1.06 to 1.07)	< 0.001
10-year follow-up					
HR (95%CI)	Ref	2.37 (2.13 to 2.64)	4.77 (3.54 to 6.44)	1.06 (1.05 to 1.06)	< 0.001
Adjusted HR (95%CI)^b^	Ref	2.02 (1.79 to 2.27)	3.89 (2.85 to 5.31)	1.05 (1.05 to 1.06)	< 0.001

Abbreviations: HR, hazard ratio; CI, confidence interval; ESCC, esophageal squamous cell carcinoma.

a. Risk score groups were defined based on the lower and upper tertiles of risk scores.

b. Adjusting for sex (male/female), education level, and nutrition intervention groups.

## Data Availability

The datasets used and/or analysed during the current study are available from the corresponding author on reasonable request.

## Abbreviations

AIC: Akaike Information Criterion
AUC: Area Under the Curve
BMI: Body Mass Index
CHCAMS: Cancer Hospital Chinese Academy of Medical Sciences
CI: Confidence Interval
EC: Esophageal Cancer
EAC: Esophageal Adenocarcinoma
ESCC: Esophageal Squamous Cell Carcinoma
HR: Hazard Ratio
NCI: National Cancer Institute
PAHs: Polycyclic Aromatic Hydrocarbons
ROC: Receiver Operating Characteristic
TSNAs: Tobacco-Specific Nitrosamines
